# The association between fluoroquinolones and aortic dissection and aortic aneurysms: a systematic review and meta-analysis

**DOI:** 10.1038/s41598-021-90692-8

**Published:** 2021-05-26

**Authors:** Ian Wee, Brian Chin, Nicholas Syn, Keng Siang Lee, Jun Jie Ng, Andrew M. T. L. Choong

**Affiliations:** 1 SingVaSC, Singapore Vascular Surgical Collaborative, Singapore, Singapore; 2 Yong Loo Lin School of Medicine, National University of Singapore, Singapore, Singapore; 3 National University Hospital, Singapore, Singapore; 4 Bristol Medical School, University of Bristol, Bristol, UK; 5 Division of Vascular Surgery, National University Heart Centre, Level 9, NUHS Tower Block, 1E Kent Ridge Road, Singapore, 119228 Singapore; 6 Department of Surgery, Yong Loo Lin School of Medicine, National University of Singapore, Singapore, Singapore; 7 Cardiovascular Research Institute, National University of Singapore, Singapore, Singapore

**Keywords:** Cardiovascular diseases, Risk factors, Drug safety

## Abstract

Previous studies have drawn causal associations between fluoroquinolone use and collagen pathologies including tendon rupture and retinopathy. This meta-analysis

attempted to assess the association between fluoroquinolone use and the risk of aortic dissection or aortic aneurysm. A systematic search was performed on Medline, EMBASE, and the Cochrane library. 9 studies were included in final analysis. Primary random-effects meta-analysis of 7 studies, excluding 2 pharmacovigilance studies demonstrated statistically increased odds of aortic dissection (OR, 2.38; 95% CI, 1.71–3.32) aortic aneurysm (OR, 1.98; 95% CI, 1.59–2.48), and aortic aneurysm or dissection (OR, 1.47; 95% CI, 1.13–1.89; I^2^ = 72%) with current use of fluoroquinolones compared to their nonuser counterparts. Based on the “number needed-to-harm” analysis, 7246 (95% CI: 4329 to 14,085) patients would need to be treated with fluoroquinolones for a duration of at least three days in order for one additional patient to be harmed, assuming a population baseline incidence of aortic dissection and aneurysm rupture to be 10 per 100,000 patient-years. With strong statistical association, these findings suggest a causal relationship, warranting future research to elucidate the pathophysiological and mechanistic plausibility of this association. These findings however, should not cease prescription of fluoroquinolones, especially when clinically indicated.

## Introduction

Fluoroquinolones rank amongst the topmost frequently used antibiotics^1–3^. Epidemiological studies have shown an increased risk of tendon disorders associated with fluoroquinolone use, such as tendinopathy and Achilles tendon rupture^4–6^. In vitro studies have demonstrated the mechanistic associations attesting the properties of these antibiotic involved in potentiating the activity of matrix metalloproteinases, resulting in degradation of collagen and structural components of the extracellular matrix. This is accompanied by features of oxidative stress in tendon cells, and reduction of collagen production ^7–10^.

Collagen constitutes a major extracellular matrix component of the aortic wall, particularly of variants type I and III^11^, raising the concern that fluoroquinolones may increase the risk of the formation of aortic aneurysms and dissections particularly given that these aortic pathologies are associated with changes in collagen structure and content^12^.

The incidence of aortic dissections and aneurysms are also increasing, potentially conferring profound life-threatening consequences to patients if left untreated^13–18^. Given the widespread use of fluoroquinolones, any plausible drug-related risk of aortic aneurysms and dissections should be investigated thoroughly. The main objective of this present study was to appraise real-world evidence of the association between fluoroquinolone and the occurrence of aortic dissections or aneurysms.

## Results

**Figure 1:**
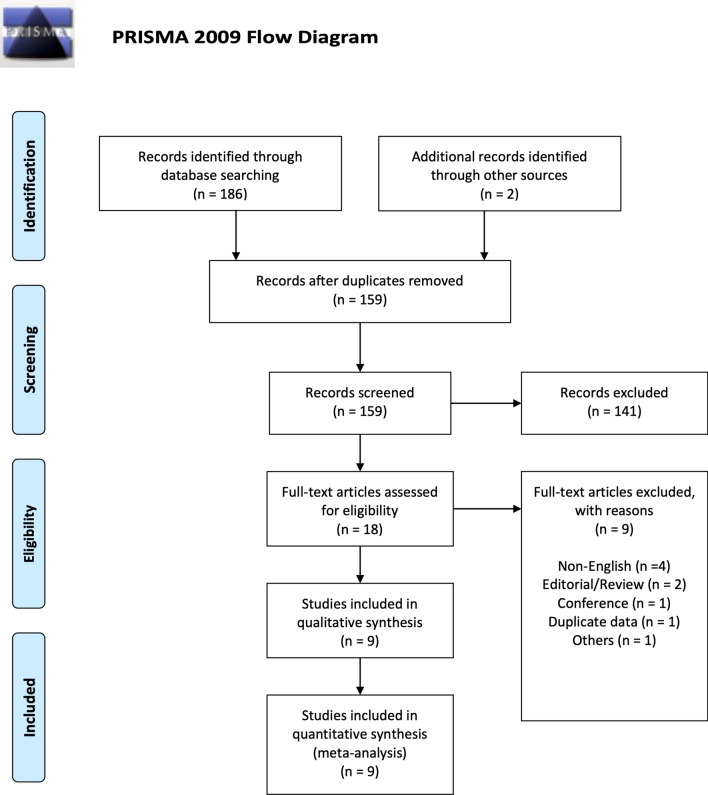
PRISMA flow diagram.

The systematic search revealed 159 unique titles, 2 additional articles were manually sourced from reference lists which also met the inclusion criteria. 18 were included after title and abstract screening, and reviewed in their entirety. Nine were excluded due to various reasons as shown in Fig. 1 (colored), leaving 9 studies in the final analysis^19–27^. The risk of bias assessments can be found in Tables 3 and 4.

### Study design

Characteristics of all 9 studies are shown in Table 1. Daneman et al.’s population-based longitudinal cohort study incepted 1.7 million elderly participants (aged above 65 years old) from the Ontario Registered Persons Database between 1997 and 2012, with a total follow-up duration of 22,380,515 patient-days on treatment. Lee et al.’s propensity-matched population-based nested case control study included 1477 cases and 147,700 matched controls (based on age, sex, index date) from the National Health Insurance Research Database in Taiwan. Pasternak et al.’s propensity-matched population-based cohort study incepted a cohort of 360 088 patients (aged above 50 years old) with fluoroquinolone use from linked nationwide data from Swedish registers 2006–2013, subsequently compared with another 360,088 propensity score-matched comparator episodes of amoxicillin use. All 9 studies evaluated different types of fluoroquinolones, with Taiwan studies by Dong et al. and Lee et al. evaluating additional fluoroquinolones unavailable in North America. In addition, both Daneman and Lee et al.’s studies had multivariable adjustment for demographic cardiovascular and healthcare use including indications for fluoroquinolone usage and comorbidity score.

**Table 1:** Study design of included studies.

Study Authors	Study Design	Country	Follow-up Duration	Participants	Interventions	Selection Criteria	Controls	Main Outcomes
Daneman et al	Population-based longitudinal cohort study	Ontario	Patient-days on treatment: 22,380,515	Inception cohort of > 65y from Ontario Registered Persons Database 1997–2012	Ciprofloxacin, levofloxacin, ofloxacin, norfloxacin, moxifloxacin in outpatient setting	AA and AD using administrative codes	Positive and negative tracer exposure	Tendon rupture, retinal detachment, AA and AD within a 30 day risk window after treatment
Dong et al	Population-based nested case control	Taiwan	Mean Follow-up Duration: 1303.82 [723.12] days	Adult patients from the Taiwan population-based health insurance claims database from 2009–2015	Fluoroquinolones	Cases of first AA and AD requiring hospitalization using the ICD-9-CM code 441 in any diagnosis position for the cases	Each incident case of AA/AD was matched with 10 control individuals by age, sex, and follow-up duration in the database using risk-set sampling	Tendon rupture, retinal detachment, AA and AD within a 60 day at risk window after treatment
Gopalakrishnan et al	Propensity-matched population-based cohort study	United States	60 days	Adult patients from United States commercial health insurance claims database from 2003–2015	Ciprofloxacin, levofloxacin	139,772 treatment episodes of fluoroquinolone use	139,722 propensity score-matched comparator episodes azithromycin use	AA and AD within a 60 day at risk window after treatment
Lee et al	Propensity-matched population-based nested case control	Taiwan	Mean Follow-up Duration: 3613.3	Adults from the Taiwan National Health Insurance Research Database 1998–2011	Ciprofloxacin, levofloxacin, ofloxacin, sparfloxacin, norfloxacin, lomefloxacin, moxifloxacin, gemifloxacin, exoxacin, pefloxacin	1477 cases of first AA or AD requiring hospitalization plus imaging with echocardiography, magnetic resonance imaging, or angiography	147,700 matched control cases	AA and AD within a 60 day at risk window after treatment
Lee et al	Case-crossover study	Taiwan	60 days	Adults from the Taiwan National Health Insurance Research Database 2001–2011	Ciprofloxacin, levofloxacin, ofloxacin, sparfloxacin, norfloxacin, lomefloxacin, moxifloxacin, gemifloxacin, exoxacin, pefloxacin	1213 cases of first AA or AD requiring hospitalization plus imaging with echocardiography, magnetic resonance imaging, or angiography	Participants acted as their own control	AA and AD within a 60 day at risk window after treatment
Maumus-Robert et al	Case-time control-study	France	180 days	Adult patients from the French health insurance nationwide databases from 2010–2015	Fluoroquinolones	Incident aortoiliac aneurysm or dissection who were diagnosed between 2010–2015 on fluoroquinolones	Those on amoxicillin	AA and AD within a 180 day at risk window after treatment
Pasternak et al	Propensity-matched population-based cohort study	Sweden	Patients contributed person time from the date a prescription was filled, to the date of an outcome event, end of follow-up (120 days), end of study period, hospital admission, death, or new prescription for a study antibiotic	Inception cohort of > 50y from linked nationwide data from Swedish registers 2006–2013	Ciprofloxacin, norfloxacin, and other fluoroquinolones	360 088 treatment episodes of fluoroquinolone use	360 088 propensity score-matched comparator episodes of amoxicillin use	AA and AD within a 60 day at risk window after treatment
Meng et al	Pharmacovigilance study	China		Adult patients from the US Food and Drug Administration Adverse Event Reporting System (FAERS) 2004–2016	Ciprofloxacin, levofloxacin and moxifloxacin	7153 801 adverse event reports: 2713 for aortic aneurysms and 1008 for aortic dissections	Those on cefuroxime	AA and AD at risk window after treatment (timeframe not specified)
Sommet et al	Pharmacovigilance study	France		Patients > 50y registered on the Vigibase, World Health Organization Global Individual Case Safety Reports (ICSRs) database from 1972 to 2017	Levofloxacin, Ciprofloxacin, Moxifloxacin, Ofloxacin, Gatifloxacin, Tosufloxacin, Enoxacin, Fleroxacin, Gemifloxacin, Grepafloxacin, Lomefloxacin, Norfloxacin, Pazufloxacin, Pefloxacin, Prulifloxacin, Rufloxacin, Sparfloxacin, Temafloxacin, Trovafloxacin	172,588 were reported with fluoroquinolones	40,658 with amoxicillin	Tendonitis, tendon rupture, AA and AD at risk window after treatment (timeframe not specified)

### Exposure

Fluoroquinolone exposure was defined differently in most studies according to various prescription databases but range from 60 to 180 days. For instance, Daneman’s cohort study defined exposure risk as up to 30 days after fluoroquinolone use. Lee’s case control study categorised exposures into current fluoroquinolone exposure within 60 days of index date, recent exposure within 60–365 days before index date, and any exposure for 3 or more days in the 1-year period before index date. Pasternak’s cohort study defined exposure to fluoroquinolones as a 60-day period from start of treatment, with secondary analysis investigating subsequent 60 days (days 61 to 120). Further subdivision of the 60-day risk period into 10 day intervals was conducted to assess number of events per interval. Both pharmacovigilance studies by Meng and Sommet did not provide a timeframe for the at risk window.

### Outcome

3 studies utilised administrative diagnostic codes to identify aortic aneurysms and dissections. The primary outcomes were collagen-associated severe toxicities in Daneman’s study, and first occurrence of aortic aneurysm or dissection in inpatients in both Lee’s and Pasternak’s study.

### Primary meta-analysis

**Figure 2:**
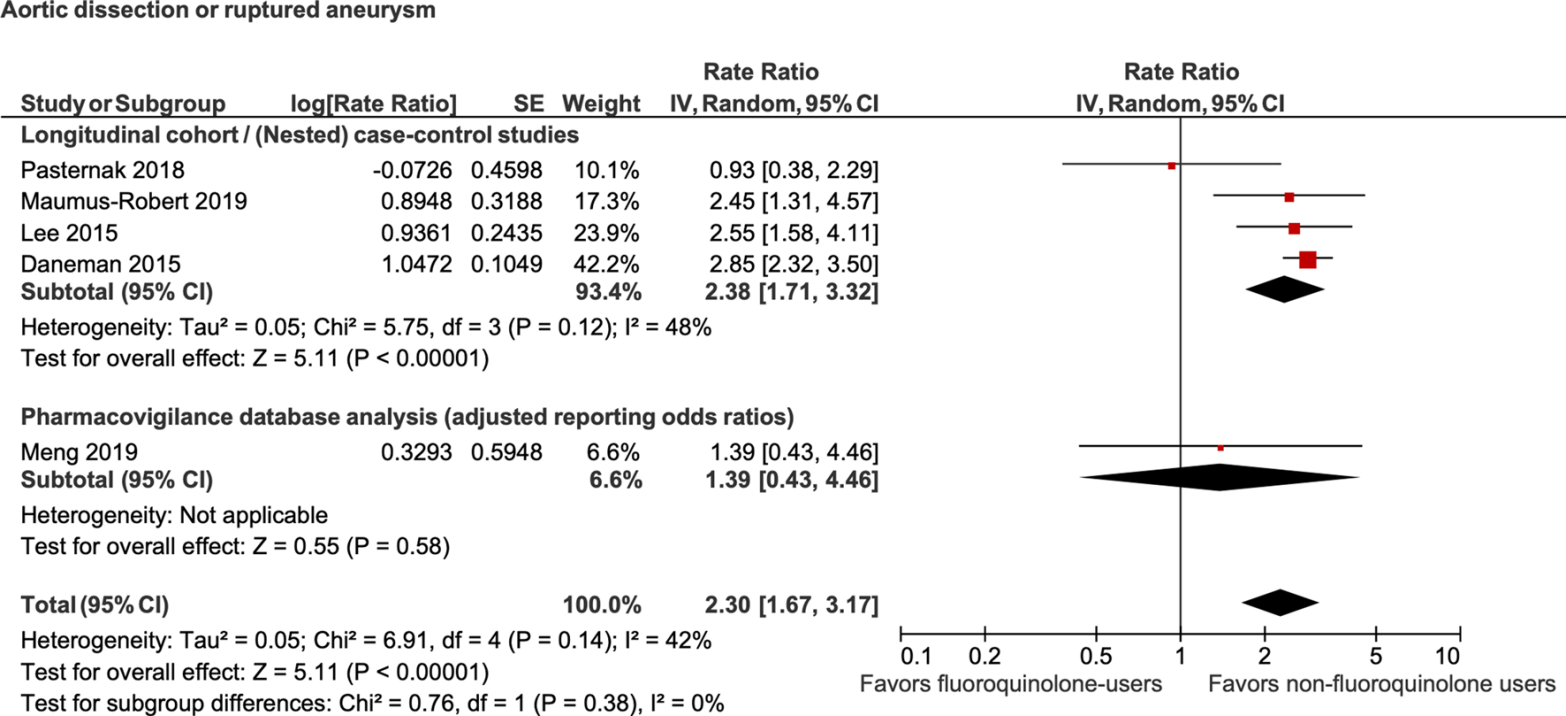
Association between fluoroquinolone use and aortic dissection.

**Figure 3:**
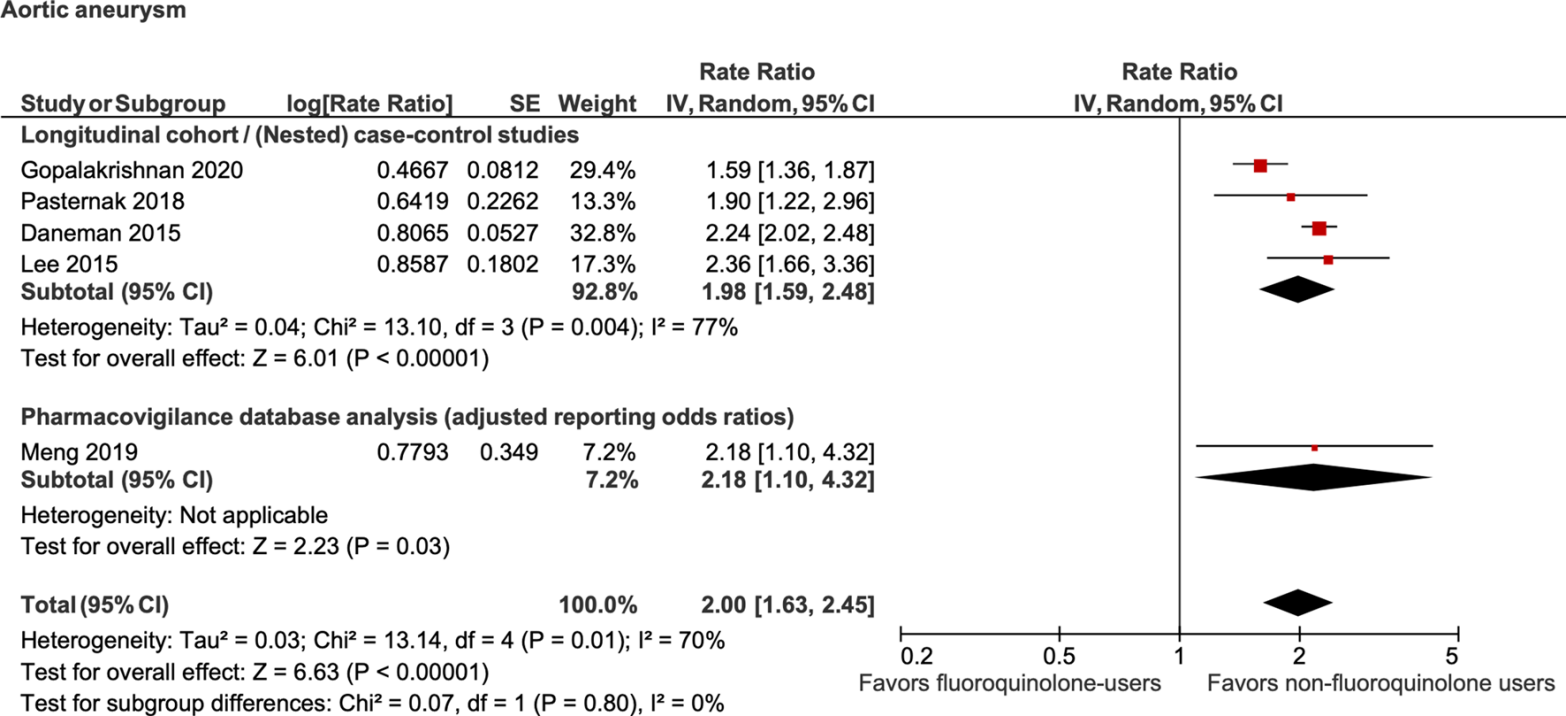
Association between fluoroquinolone use and aortic aneurysm.

**Figure 4:**
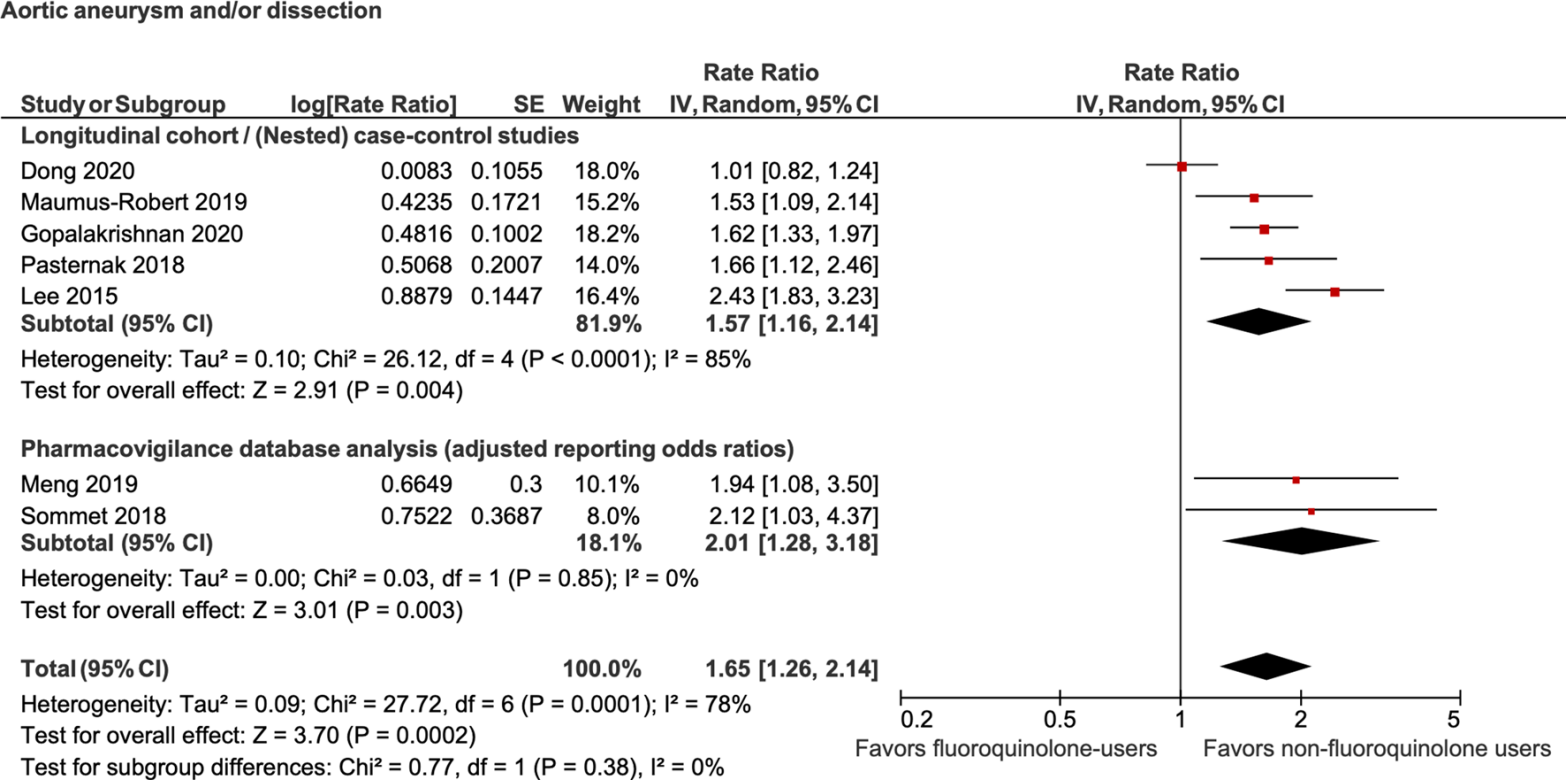
Association between fluoroquinolone use and aortic dissection or aortic aneurysm.

Random-effects meta-analysis of 4 studies reflected that current use of fluoroquinolones was associated with a statistically significant increased odds of aortic dissection (OR, 2.38; 95% CI, 1.71–3.32; *P* < 0.00001; I^2^ = 48%). Meta-analysis of another 4 studies reflected that current use of fluoroquinolones was associated with a statistically significant increased odds of aortic aneurysm (OR, 1.98; 95% CI, 1.59–2.48; *P* < 0.00001; I^2^ = 77%). Finally, meta-analysis of 5 studies showed a significantly increased odds of aortic dissection or aneurysm (OR, 1.57; 95% CI, 1.16–2.14; *P* = 0.004; I^2^ = 85%) with current use of fluoroquinolones compared to their nonuser counterparts (Figs. 2, 3 and 4—colored). The results of this primary analysis excluded the 2 pharmacovigilance studies and is considered more robust, because of greater similarity between the cohort & case–control studies as compared to the pharmacovigilance studies (the patient populations of the pharmacovigilance database represent a selected population of patients who were reported to the FDA for experiencing an adverse drug event).

### Sensitivity analysis

A sensitivity analysis was performed by including the full list of included studies including the pharmacovigilance studies. Random-effects meta-analysis of 5 studies reflected that current use of fluoroquinolones was associated with statistically significant increased odds of aortic dissection (OR, 2.30; 95% CI, 1.67–3.17; *P* < 0.00001; I^2^ = 42%). Meta-analysis of another 5 studies reflected that current use of fluoroquinolones was associated with statistically significant increased odds of aortic aneurysm (OR, 2.00; 95% CI, 1.63–2.45; *P* < 0.00001; I^2^ = 70%). Finally, meta-analysis of 7 studies showed a significantly increased odds of aortic dissection or aneurysm (OR, 1.65; 95% CI, 1.26–2.14; *P* < 0.0002; I^2^ = 78%) with current use of fluoroquinolones compared to their nonuser counterparts (Figs. 2, 3 and 4).

## Hazard ratios for past exposure

Lee et al.’s study was the sole study which compared risks of aortic aneurysm and dissection between individuals with past exposure/any prior users of fluoroquinolones, and nonusers. Propensity score-matched estimates for past exposure (incidence rate ratio, 1.19; 95% CI, 0.85–1.66) and any prior users (incidence rate ratio, 1.37; 95% CI, 1.04–1.79), as well as covariate adjusted estimates for past exposure (incidence rate ratio, 1.49; 95% CI, 1.18–1.90) and any prior users (incidence rate ratio, 1.69; 95% CI, 1.39–2.06) were found to be statistically significant.

### Number needed to harm for aortic dissection or aneurysm with or without rupture

The number needed to treat to harm for the various aortic pathologies is summarized in Table 2. A range of estimates regarding baseline incidence are provided below for ease of reference to clinicians, who should tailor their decisions accordingly. For example, assuming that the population baseline incidence of aortic dissection and aneurysm rupture was 10 per 100,000 patient-years, then 7246 (95% CI: 4329 to 14,085) patients would need to be treated with fluoroquinolones for a duration of at least three days in order for one additional patient to be harmed. Even if we assumed the baseline rate of aortic dissection and aneurysm rupture to be higher at 150 events per 100,000 patient year (e.g., in high-risk patient populations such as elderly males), the number needed to harm is 483 (95% CI: 289 to 939) (Table 2).

**Table 2:** Number needed to treat to harm for aortic pathologies.

Aortic pathology (assumed rate ratio)	Assumed baseline incidence of aortic pathology (number of events per 100,000 patient years) Note: baseline incidence may vary across geography, gender, age, and other clinical characteristics. A range of estimates regarding baseline incidence are provided below for ease of reference to clinicians, who should tailor their decisions accordingly	Number needed to harm (NNH) (95% CI)
**Aortic dissection or aneurysm rupture** (Pooled rate ratio: 2.38 [95% CI 1.71 to 3.32])	3	24,155 (95% CI: 14,430 to 46,948)
	5	14,493 (95% CI: 8658 to 28,169)
	10	7246 (95% CI: 4329 to 14,085)
	15	4831 (95% CI: 2886 to 9390)
	25	2899 (95% CI: 1732 to 5634)
	50	1449 (95% CI: 866 to 2817)
	75	966 (95% CI: 577 to 1878)
	100	725 (95% CI: 433 to 1408)
	125	580 (95% CI: 346 to 1127)
	150	483 (95% CI: 289 to 939)
**Aortic aneurysm** (Pooled rate ratio: 1.98 [95% CI 1.59 to 2.48])	5	20,408 (95% CI: 13,514 to 33,898)
	25	4082 (95% CI: 2703 to 6780)
	50	2041 (95% CI: 1351 to 3390)
	75	1361 (95% CI: 901 to 2260)
	100	1020 (95% CI: 676 to 1695)
	125	816 (95% CI: 541 to 1356)
	150	680 (95% CI: 450 to 1130)
	250	408 (95% CI: 270 to 678)
	300	340 (95% CI: 225 to 565)

### Risk of bias and publication bias

The risk of bias was assessed using the Newcastle–Ottawa scale. Overall, the risk of selection and exposure bias were low. However, the overall comparability was deemed to be inadequate because the studies failed to control for all important risk factors of aortic aneurysm and dissection, particularly smoking. The assessment of publication bias was not meaningful due to the small number of studies, and hence was not performed. Results of the assessments can be found in Tables 3 and 4.

**Table 3:** Risk of bias assessment for case–control studies.

First author, year	Selection				Comparability	Exposure			Total points (/9)
	Adequate case definition	Representativeness of the cases	Control selection	Definition of controls	Comparability of Cases and Controls on the Basis of the Design or Analysis	Ascertainment of exposure	Non-response rate	Similar method of ascertainment for cases and control	
Dong 2020	Yes	Yes	Community controls	No history of disease	Study controls for risk factors for aortic aneurysm but lacks other important risk factors (smoking)	Medical records	N.A	Yes	7/9
Lee2015	Yes	Yes	Community controls	No history of disease	Study controls for risk factors for aortic aneurysm but lacks other important risk factors (smoking)	Medical records	N.A	Yes	7/9
Lee2018	Yes	Yes	Community controls	No history of disease	Study controls for risk factors for aortic aneurysm but lacks other important risk factors (smoking)	Medical records	N.A	Yes	7/9
Maumus-Robert 2019	Yes	Yes	Community controls	No history of disease	Study controls for risk factors for aortic aneurysm but lacks other important risk factors (smoking)	Medical records	N.A	Yes	7/9

**Table 4:** Risk of bias assessment for cohort studies.

First author, year	Selection				Comparability	Outcome			Total points (/9)
	Representativeness of exposed cohort	Selection of non-exposed cohort	Ascertainment of exposure	Demonstration that outcome of interest was absent at start of study	Comparability of Cohorts on the Basis of the Design or Analysis	Assessment of Outcome	Length of follow-up	Adequacy of follow up of cohorts	
Daneman2015	Elderly aged 65 years old and above	Same source as exposed cohort	Ontario database	Adequate	Inadequate (did not control for other important risk factors for aortic aneurysm and aortic dissection)	Record linkage	Adequate	Complete	7/9
Gopalakrishnan2020	Patients aged 50 years old and above	Same sources as exposed cohort	United States database	?Adequate	Inadequate (did not control for other important risk factors for aortic aneurysm and aortic dissection)	Record linkage	Adequate	Complete	7/9
Pasternak2018	Representative	Same sources as exposed cohort	Swedish registers	Adequate	Inadequate (did not control for other important risk factors for aortic aneurysm and aortic dissection)	Record linkage	Adequate	Complete	7/9

## Discussion

There is moderate evidence from this meta-analysis demonstrating the heightened risk of aortic aneurysm or aortic dissection in patients treated with fluoroquinolones. We believe, to our knowledge, this to be the first meta-analysis including the highest number of studies analyzing this relationship. Furthermore, we have performed additional analysis to comprehensively generate the numbers needed to harm based on a wide spectrum of assumed population baseline incidences of aortic aneurysms and dissections.

These findings may be supported by biological plausibility, where fluoroquinolones have been shown to induce epigenetic changes, and augment collagen maturation^28^. Chief of all molecular players is matrix metalloproteinases (MMPs), where its dysregulation propagates extracellular matrix degeneration^29–31^. Cellular studies of smooth muscle cells derived from aortic aneurysms demonstrate an increased expression of MMP-9 and MMP-2^32^, both of which potentiate collagenolytic activity^33, 34^. These corroborated with animal studies showing an increased expression of MMP-9 and MMP-2 in cornea tissue^35^ and MMP-2 in tendon tissue^36^ upon administration of ciprofloxacin. Hence, the destruction of collagen and connective tissue by fluoroquinolones in the aortic wall might portend the development of aortic aneurysm and dissection.

An important temporal aspect of our findings is the timing of this association, where the risk of aortic dissection and aortic aneurysm was increased with current use of fluoroquinolones. Two studies defined this as 60 days after start of treatment^20, 21^; one study defined it as 30 days after start of treatment^19^. In particular, Pasternak and colleagues^21^ propounded this association to be the most significant in the first 10 days post-treatment, suggesting that this phenomenon might occur acutely during the active treatment phase. Albeit preliminary, this finding is consistent with in vitro studies demonstrating prompt expression of MMPs in tendon cells after ciprofloxacin administration^8, 36^. The seemingly acute nature of the risk period is observed in other pathologies. For instance, fluoroquinolone-induced tendon rupture occurred between 2 and 31 days with a median of 7 days^37^. Similarly, the highest risk of fluoroquinolone-associated retinal detachment was within 5 days of treatment^38^.

However, our findings must be interpreted in the context of known limitations. First, there were no randomized controlled trials in this study. However, it is not entirely true that observational studies have no role in exemplifying a causal association between an exposure and outcome. Moreover, it is unlikely there will ever be a randomized human trial investigating the association between fluoroquinolone exposure and aortic pathology from practical and ethical standpoints. Only one study compared risks of aortic aneurysm and dissection between individuals with past exposure/any prior users of fluoroquinolones, and nonusers^20^. One limitation of the available data is that there is little known about the impact of past exposure or whether there is a cumulative dose effect, and hence we were unable to weight the doses of historical doses versus current doses differently. Next, confounding factors are inevitable in any observational study. Although the original studies provided adjusted estimates for a spectrum of possible confounders employing propensity score method, this does not eliminate residual confounders including smoking status and other cardiovascular risk factors. Moreover, two^19, 20^ out of three studies only adjusted for a limited number of covariates, hence potentially missing out several other potential confounding factors. In addition, there could be a possibility of protopathic bias, where clinical symptoms of aortic aneurysm and dissection are misinterpreted as infection, necessitating antibiotics treatment. However, this appears unlikely given the low risk of such misdiagnosis in real-life settings^39^. In addition, there could be a risk of detection bias, as elegantly pointed out by Lee and colleagues^20^. Given the propensity of imaging examinations such as computed tomographic scans ordered during acute infections, this increases the probability of newly diagnosed aortic pathologies. One other methodological flaw consistent in the original studies was the lack of an active comparator. Whilst Pasternak and colleagues^21^ used amoxicillin as an active comparator to limit confounding factors associated with indication (infection), two other studies compared fluoroquinolone use with no use of antibiotics^19, 20^.

Although several factors lend support to the strength of the association, including biological plausibility; consistent epidemiological evidence; and analogous findings from other pathologies, further research is warranted in other populations to establish a firm conclusion. Furthermore, it is unclear whether the association is driven by aortic aneurysm or aortic dissection, although Pasternak and colleagues argued it could be the former^21^. A limitation of our meta-analysis is that cardiovascular risk factors were not controlled for uniformly in the studies included in the analysis. In this light, we propound that this relationship cannot be proven causal given the aforementioned. Nonetheless, clinicians should continue to prescribe fluoroquinolones if clinically indicated, and we are not advocating cessation of their used based on a theoretical risk of aortic dissection or aortic aneurysm development. Further evidence is warranted to accurately elucidate the pathophysiology of how this may occur mechanistically. With strong statistical association, our findings suggest a causal relationship between the use of fluoroquinolone and increased risk of aortic aneurysm and dissection. Further research is needed to corroborate with these current findings. However, given the numbers needed to harm, this should not change the contemporary evidence-based practice on the use of fluoroquinolone. This information should be used for purposes of patient counselling. If the patient is met with a condition that has alternative suitable antibiotic of same efficacy these options should be discussed.

## Methods

This review was performed in accordance to the Preferred Reporting Items for Systematic Reviews and Meta-Analyses (PRISMA) statement guidelines^40^. The study protocol was registered on the PROSPERO International Prospective Register of Systematic Reviews (registration number CRD42020212150).

### Eligibility criteria

Any randomized or non-randomized study (cohort study; case–control study) that investigated the association between fluoroquinolone use regardless of indication, and risk of aortic dissection and/or aortic aneurysm were included. The controls were limited to unexposed patients or patients receiving other types of antibiotics. The following designs were excluded: case reports/series; non-English; animal studies. Studies that did not report extractable data including odds ratio; relative risk; hazard ratio; or raw data, were excluded. Conference abstracts were considered only if sufficient data was available for meaningful synthesis and analysis.

### Outcome measures

The primary outcomes of interest were development of aortic dissection or aortic aneurysm amongst patients who: 1) have taken fluoroquinolones in the last 1 year; 2) are currently taking fluoroquinolones for active treatment, limited up to 60 days within index date.

### Systematic search

A systematic search was performed in the following databases: Medline; EMBASE; and the Cochrane library from inception on 30th September 2020. An exhaustive combination of the following ‘MeSH’ and ‘non-MesH’ terms were used: aortic dissection; aortic aneurysm; fluoroquinolones; quinolones. The full search strategy is available upon request from IW.

### Selection of studies and data extraction

Three reviewers (IW, BC, KSL) independently screened and assessed the studies for potential inclusion in two stages: (1) title and abstract screen; (2) full-text review of those titles included in the first stage. Data extraction was also performed independently by three reviewers (IW, BC, KSL) using a standardized proforma. The following data were abstracted: study design, demographics, country and dataset, type of fluoroquinolones, indication for fluoroquinolones, selection criteria, controls, unadjusted hazard ratios (HR) or odds ratio (OR), propensity-score adjusted HR, propensity-score matched HR, and covariate-adjusted HR. Conflicts were resolved by consensus, or by appeal to a fourth senior author (AC).

For studies with outcomes of interest reported but not specific to fluoroquinolone exposure, the corresponding authors were contacted for acquisition of raw data. A response was anticipated for two weeks before a decision was made on the eligibility of the study.

The reference lists of included studies were also scrutinized to pursue references of identified citations, in an effort to identify high quality resources in obscure locations that could have been overlooked in our search strategy^41^.

### Risk of bias and quality assessment

The Newcastle–Ottawa Scale for Quality Assessment for cohort and case–control studies was employed to assess the risk of bias of included studies^42^. In brief, the assessment of case–control studies includes 8 domains: (1) adequate case definition; (2) representativeness of cases; (3) control selection; (4) definition of controls; (5) comparability of cases and controls on the basis of design or analysis; (6) ascertainment of exposure; (7) non-responses rate; (8) similar method of ascertainment for cases and control. The assessment of cohort studies also includes 8 domains: (1) selection of non-exposed cohort; (2) representativeness of cases; (3) ascertainment of exposure; (4) demonstration that outcome of interest was absent at start of study; (5) comparability of cohort on the basis of design or analysis; (6) assessment of outcome; (7) length of follow-up; 8) adequacy of follow-up of cohorts. Based on the aforementioned, the studies will be scored against a maximum of 9 points.

### Data analysis

Meta-analyses were done assuming the random effects model to account for both within-study and between-study heterogeneity. The random-effects meta-analysis was employed even when quantitative heterogeneity was low (defined as having an I^2^ statistic value of less than 50%), in order to also account for qualitative heterogeneity which may not have been evident from the I^2^ value alone. Conditional odds ratios (ORs) (which was the effect measure in these studies)^20, 23, 24^ were taken to be interchangeable with the hazard ratios since the likelihood functions of the conditional logistic model is mathematically identical to the stratified Cox model when estimated under partial likelihood. The incidence rate ratios and hazard ratios were likewise taken to approximate one another owing to the functional similarities between the Poisson and Weibull regressions, while the relative risk was assumed to approximate the hazard ratio since right-censoring due to loss to follow-up was likely to be minimal because the exposure-time and follow-up duration were short. As such, the main effect measure in our analyses was the incidence rate ratio.

For the study by Meng et al.^22^ we calculated the “cefuroxime-adjusted” reporting odds ratio (ROR) by taking the ratio of the pooled ROR of the three fluoroquinolones divided by the ROR of cefuroxime (the negative control). Notably, this approach is similar to that of the study by Maumus-Robert et al.^23^ which reported an “amoxicillin-adjusted” odds ratios, where Maumus-Robert et al. calculated as the ratio of ORs for fluoroquinolones divided by OR for amoxicillin). The 95% confidence intervals for the adjusted reporting ORs were calculated assuming a lognormal distribution for the adjusted OR, as follows:

$${\text{Point\,estimate\,of\,adjusted}}ORs \, = \, AOR \, = \, OR_{{{\text{fluoroquinolone}}}} /OR_{{{\text{control}}}}$$

$${\text{Standard error}}\left[ {OR_{{{\text{fluoroquinolone}}}} } \right] \, = \, (\ln \left( {UCL_{{{\text{fluoroquinolone}}}} {-} \, LCL_{{{\text{fluoroquinolone}}}} } \right)/1.96.$$

$${\text{Standard}}\;{\text{error}}\left[ {OR_{{{\text{control}}}} } \right] \, = \, (\ln \left( {UCL_{{{\text{control}}}} {-} \, LCL_{{{\text{control}}}} } \right) \, / \, 1.96.$$

The pooled standard error can be calculated as:

$${\text{SEpooled }} = \, sqrt\left[ {({\text{Standard}}\;{\text{error}}} \right[{\text{ORfluoroquinolone}}\left] {)^{2} + \, ({\text{Standard}}\;{\text{error}}} \right[{\text{ORcontrol}}\left] {)^{2} )} \right]$$

The 95% confidence intervals of the AOR can then be calculated as: exp[ln(AOR) + /- 1.96 * SEpooled].

For example, the Maumus-Robert study reported an OR of fluoroquinolones of 3.23 (95% CI: 1.85 to 5.64) and an OR of amoxicillin of 1.32 (95% CI: 0.99 to 1.76)^23^. The Maumus-Robert study also reported an amoxicillin-adjusted OR of 2.44 (95% CI: 1.31 to 4.57)^23^. Using our formula above, we are able to reverse-engineer the adjusted OR, obtaining an amoxicillin-adjusted OR of 2.45 (95% CI: 1.31 to 4.58). Notably, our estimate of the adjusted OR is nearly identical to that of the exact OR provided by Maumus-Robert et al.^23^ Hence, these formulae were likewise applied to the Meng 2019 study to estimate the cefuroxime-adjusted OR.

The number needed to treat to harm, and its 95% CI was also assessed to estimate an absolute measure of effect. The number needed to treat to harm is the number of patients who need to be treated with fluoroquinolones for 1 additional patient to have an adverse event. The baseline risk was obtained from the unexposed group in the population-based studies.

### Ethical approval for research

Ethical approval was not required for this systematic review and meta-analysis.

## Supplementary Information

Supplementary Information.
